# Extra-large G proteins and their roles in plant development and responses to environmental stresses

**DOI:** 10.3389/fpls.2026.1823314

**Published:** 2026-05-05

**Authors:** Shweta Meshram, Oluwatoyosi F. Akintayo, Madan K. Bhattacharyya

**Affiliations:** 1 Department of Agronomy, Iowa State University, Ames, IA, United States; 2 Biologicals Lab, Big Biological Pioneer Pvt. Ltd, BSCBioNEST Incubator, Regional Center For Biotechnology, Faridabad, India

**Keywords:** extra-large GTP-binding protein (XLG), G-proteins, plant pathogens, plant signaling, stress response, XLG3 gene

## Abstract

Eukaryotic G (guanine nucleotide-binding) proteins mediate important signal transduction pathways involved in diverse biological processes, including plant immunity against a wide range of pathogens. Plant signaling relies significantly on G proteins, including the Gα, Gβ, and Gγ subunits, which mediate important functions. Extra-large GTP-binding proteins (XLGs) are a subfamily of plant-specific Gα proteins that structurally resemble canonical Gα proteins but have distinct functional roles. Unlike canonical Gα proteins, XLGs lack intrinsic GTPase activity and possess an extended N-terminal region with nuclear localization signals, and interact with plasma membrane-localized receptors, suggesting their roles beyond plasma membrane signaling. XLGs play an essential role in a variety of physiological processes, such as root morphogenesis, chloroplast development, and responses to biotic and abiotic stressors. Among the XLGs, XLG1, XLG2, and XLG3 play especially vital roles with XLG2 and XLG3 involved in pathogen perception and signal transduction for activation of immune responses. XLGs interact with G-protein subunits and receptor-like kinases (RLKs), particularly FLS2 and BIK1, to form dynamic complexes involved in defense signal transduction. Several recent invesitgations demonstrate the significance of XLGs in modulating plant immunity and development suggesting their application for enhancing crop output and resilience. Understanding the mechanisms behind XLG functions may assistin the development of novel approaches to enhance plant resilience to environmental challenges. This review highlights the roles of XLG proteins in plant development and stress responses.

## 1 Introduction

G protein heterotrimers, composed of the Gα, Gβ, and Gγ subunits, are essential for animal and plant signaling pathways. G proteins play pivotal roles in transducing extracellular signals into intracellular responses. They coordinate a wide array of physiological processes in yeast, plants and animals (Dohlman and Thorner, 2001; Vidhyasekaran, 2013; Maruta et al., 2021). G protein-coupled receptors (GPCRs), also known as seven-transmembrane-spanning (7TM) proteins transmit signals from the plasma membrane in animals (Tesmer, 2016). While the presence and functionality of GPCR-like receptors in plants is somewhat controversial and remain a topic of ongoing research, recent studies suggest that plant G proteins may interact with receptor-like kinases (RLKs) rather than conventional GPCRs (Pandey, 2020). This interaction model supports a unique mechanism of G-protein activation in plants, which bypasses the classical seven-transmembrane domain structure seen in animal GPCRs (Bender and Zipfel, 2018; Pandey, 2020) More work is needed to determine if the plant GPCR homologs are orthologous to animal GPCRs (Bender and Zipfel, 2018).

Plant G proteins exhibit functional diversity despite having a limited number of Gα, Gβ, and Gγ subunits compared to that in animals. While metazoans have a larger variety of G protein subunits, such as several subfamilies of Gα subunits and a broad range of regulators, the interactions of plant G proteins are distinct. This diversity is reflected in their various functional roles such as host and non-host resistance, growth regulation, hormone signaling, and stress responses (Luttrell, 2006; Gill et al., 2015; Pandey, 2020; Tiwari et al., 2021). GTPase accelerating protein such as, RGS1 (Regulator of G-protein Signaling-1) found in animals show similarity to the plant GPCR1 (G-protein Coupled Receptor-1) that are reported to modulate signaling and cellular behavior (Pandey et al., 2008; Zou et al., 2024). G proteins may also be activated without a GPCR (Urano et al., 2012; Urano and Jones, 2014).

**Figure 1:**
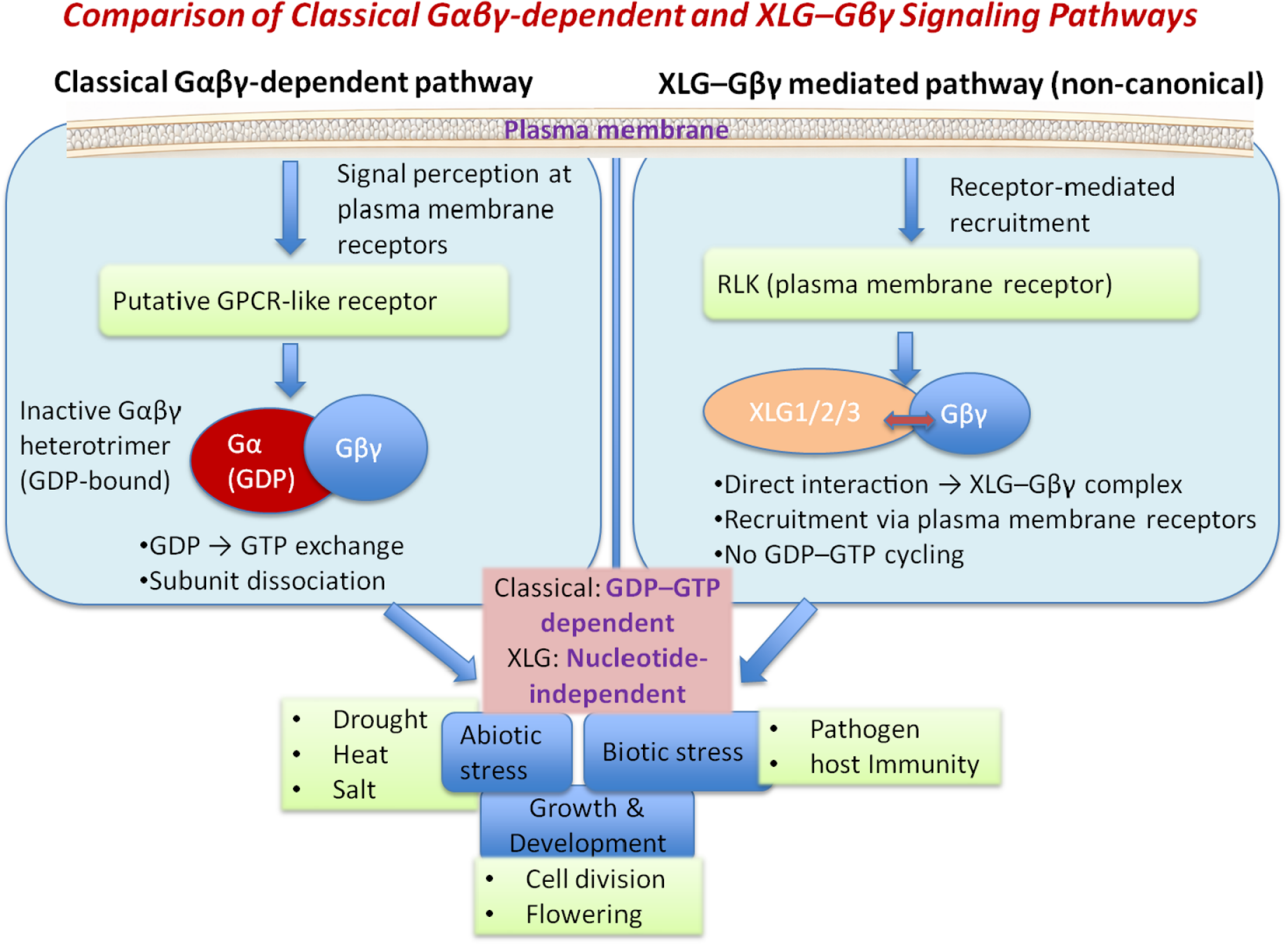
Comparison of classical Gαβγ-dependent and XLG–Gβγ signaling pathways in plants. In the classical pathway, a putative GPCR-like receptor activates the Gαβγ heterotrimer via GDP–GTP exchange, leading to subunit dissociation and downstream signaling. In contrast, XLG proteins (XLG1/2/3) are recruited by plasma membrane-localized RLKs and directly interact with Gβγ to form a functional complex, independent of nucleotide cycling. The figure highlights the key mechanistic distinction between GDP–GTP-dependent classical signaling and nucleotide-independent XLG-mediated pathways.

Among the G protein subunits, XLGs are plant-specific Gα subunits that interact with Gβγ subunits and receptor-like kinases (RLKs), forming heterotrimeric complexes while also exhibiting nuclear and cytoplasmic functions (Peskan and Oelmuller, 2000; Maruta et al., 2021). The recent studies of XLG proteins, particularly XLG2 and XLG3, have demonstrated essential roles in pathogen perception, activation of immune responses, and their ability to compensate for the absence of canonical Gα proteins under specific stress and signaling conditions (Zhang et al., 2021).Their potential interplay with other XLG subunits provides insights into our understanding of plant stress physiology and signaling pathways mediated by XLG proteins (Petutschnig et al., 2022; Oliveira et al., 2022; Tiwari et al., 2021) (**Figure 1**). In this review, we highlight the importance of XLG3 during growth, development and physiological responses to environmental stresses.

## 2 Extra-large GTP-binding proteins

**Figure 2:**
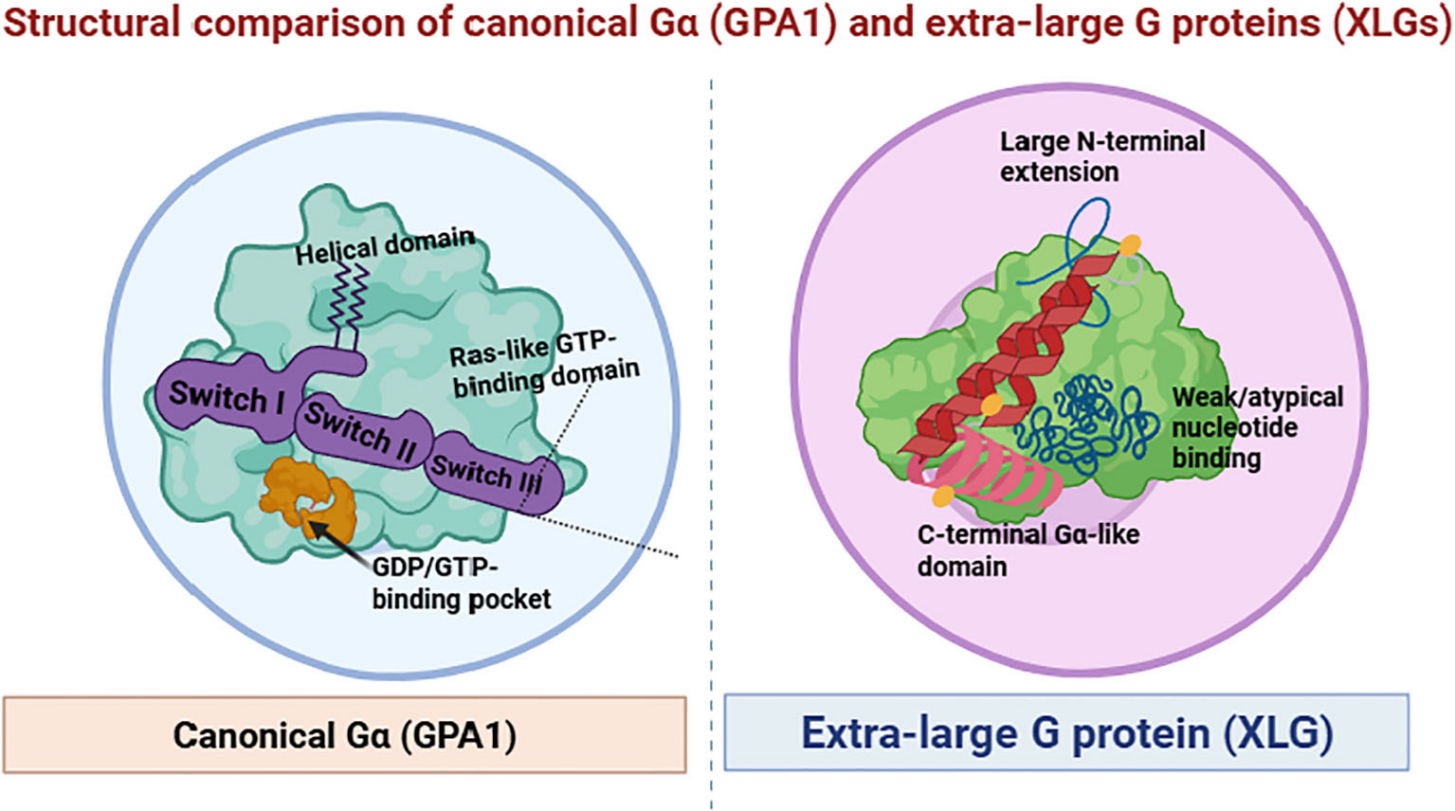
Structural comparison of canonical Gα (GPA1) and extra-large G proteins (XLGs). GPA1 contains conserved Ras-like and helical domains with defined switch regions and a GDP/GTP-binding pocket, whereas XLG proteins possess an extended N-terminal region and a divergent C-terminal Gα-like domain with weak/atypical nucleotide-binding capacity, underlying their non-canonical signaling behavior (Adapted from Maruta et al., 2015; Liang et al., 2017; Tiwari et al., 2021 and created with BioRender.com).

XLGs are atypical Gα-like guanine nucleotide-binding proteins involved in signal transduction. Unlike canonical Gα proteins, XLGs possess an extended N-terminal domain and lack efficient GTPase activity, indicating divergence in their activation and signaling pathways (**Figure 2**). They are typically involved in transmembrane signaling and intracellular modulation (Heo et al., 2012). Arabidopsis encodes three XLG proteins, XLG1, XLG2, and XLG3, which share partial sequence homology with canonical Gα proteins, particularly at their C-terminal GTP-binding domains, but exhibit unique plant-specific structural features (Ding et al., 2008; Zhu et al., 2009). These XLGs possess variable N and C terminal domains. The N-terminal domain often contains a nuclear localization signal and cysteine-rich motifs, while the C-terminal domain retains a Gα-like fold (Ding et al., 2008; Urano et al., 2016). The C-termini of the XLGs contain Gα-like domains homologous to the prototypical Gα protein (GPA1) (Ding et al., 2008), whereas the N-termini carry a cysteine-rich region and a putative nuclear localization signal (NLS) (Urano et al., 2013, 2016). Some studies describe that XLG proteins are twice the size of classical G proteins and are involved in GTP binding and hydrolysis; however, they exhibit reduced intrinsic GTPase activity compared to canonical Gα proteins, raising questions about their activation mechanism (Lee and Assmann, 1999; Heo et al., 2012). GTP binding and hydrolysis are mediated by all the three types of XLGs, XLG1, XLG2, and XLG3. However, XLGs show slow activities compared to canonical Gα subunits. In addition, Heo et al. (2012) proposed that Ca²^+^ may act as a cofactor for XLG GTPase activity; although later studies suggest that Mg²^+^ remains the primary cofactor, as is the case for canonical Gα proteins (Maruta et al., 2021). Previous studies have suggested that XLG proteins may compete with Gα, Gβ, and Gγ subunits for binding to GTP or potentially interact with heterotrimeric G-protein complexes functioning either independently or as part of atypical heterotrimers involving Gβγ, thereby modulating downstream signaling cascades (Choudhury et al., 2013; Chakravorty et al., 2015; Urano et al., 2016).

Investigations into the gene structures of XLG and canonical Gα sequences have revealed significant differences. These gene structure differences suggest evolutionary divergence and functional specialization of XLGs from classical Gα subunits (Lee and Assmann, 1999; Tiwari et al., 2021). XLG1, for example, is organized into seven exons, whereas canonical Gα genes like GPA1 have fourteen exons. The number of exons in XLG2 and XLG3 ranges from six to eight, indicating independent evolution and expansion of these genes. This variation in exon count and the divergence in intron sizes and sequences among XLG proteins suggest a complex evolutionary history (Lee and Assmann, 1999; Tiwari et al., 2021). XLGs form functional complexes with Gβγ dimers (XLG–Gβγ), which exhibit alternative G-protein signaling behaviors not seen in canonical GPA1-containing heterotrimers (Chakravorty et al., 2015; Maruta et al., 2021). XLGs are believed to have originated from a canonical Gα subunit and have retained their prototypical interaction with Gβγ dimers. In conjunction with Gβγ, XLGs play a crucial role in various processes, such as plant defense (Chakravorty et al., 2015; Patel et al., 2020). Some studies have provided insight into the relation of XLG3 with a range of heterotrimeric G protein receptors for signaling (Jose and Choudhury, 2020). The C-terminal regions of Arabidopsis XLG3, XLG1, and XLG2 show 26.1%, 23.2%, and 28.5% sequence identity with the Gα protein GPA1, respectively (Urano et al., 2016; Liang et al., 2017).

The function of a protein can be hypothesized based on its sub-cellular localization and its interactions with specific induced and/or constitutive proteins. Canonical Gα subunits like GPA1 are primarily localized to the plasma membrane. GPA1 also regulates chloroplast development, where its activation suppresses FtsH-mediated inhibition (Zhang et al., 2009). Whereas XLG proteins exhibit dual localization to the nucleus and plasma membrane, which may reflect their bifunctional roles in transcriptional regulation and membrane signaling (Ding et al., 2008; Maruta et al., 2015). Dual localization of XLG2 and XLG3 to both the nucleus and plasma membrane has been observed, particularly during pathogen attack (Adjobo-Hermans et al., 2006; Ding et al., 2008; Maruta et al., 2015; Zeng et al., 2007; Ma et al., 2022). The nuclear localization of a GFP fused XLG2 protein was observed following infection with the bacterial pathogen *Pseudomonas syringae* in *Arabidopsis* (Zhu et al., 2009). This supports the hypothesis that XLGs might serve as cytoplasmic-nuclear signal transducers in stress conditions. The nuclear localization of XLG proteins in *Arabidopsis* and tobacco has been reported (Ding et al., 2008; Chakravorty et al., 2015 and Liang et al., 2017). The canonical Gα protein primarilylocalized to the plasma membrane. Gβ is also predominantly localized to the plasma membrane, where it forms complexes with Gα and Gγ subunits. It has also been observed in the *Arabidopsis* endoplasmic reticulum (Anderson and Botella, 2007; Wang et al., 2007, 2018).

## 3 Role of XLG proteins in plant development

Reverse genetics studies have led to identification of roles of XLGs in various biological functions during plant development and signaling, including cell proliferation, organogenesis, and hormone-mediated pathways (Tiwari et al., 2021). XLGs take part in the regulation of plant growth and development processes like chloroplast development, and meristem formation (Heo et al., 2012; Bommert et al., 2013; Liang et al., 2017; Pandey and Vijayakumar, 2018). XLGs have also been implicated in controlling flowering time and meristem identity through unknown mechanisms (Liang et al., 2017; Tiwari et al., 2021) *Arabidopsis* XLGs also participate in the basic physiological functions, such as organ formation, and the regulation of root morphogenesis through cell proliferation, particularly in root apical meristems and lateral root primordia (Chakravorty et al., 2015). All three XLG genes are expressed throughout the plant, with particularly strong expression in vascular tissues, root meristems, and developing lateral roots (Pandey et al., 2008; Roy Choudhury et al., 2020).

Investigation of T-DNA insertional mutants revealed functional redundancies among the XLGs with *xlg1xlg2xlg3* triple mutant exhibiting severe phenotypes than either single or double mutants (Ding et al., 2008).The *xlg1xlg2xlg3* mutant showed changes in responses to sugar, ABA, and ethylene indicating hormone cross-talk could be regulated by XLGs (Ding et al., 2008). G proteins, including XLGs, are essential for sporophyte development and successful lifecycle completion in bryophytes such as *Physcomitrella patens* (Hackenberg et al., 2016).

A study conducted on a lower model plant, *Physcomitrella patens* lacking the canonical Gα protein, confirmed that XLGs can substitute Gα in forming functional heterotrimeric complex (Hackenberg et al., 2016). In contrast, in higher plants such as *Arabidopsis*, XLGs and the canonical Gα subunit GPA1 function in parallel, not as direct substitutes, to promote cell proliferation and development (Urano et al., 2016). XLG and Gα interact with Gβγ dimers forming atypical heterotrimers that regulate the growth and development in higher plants (Maruta et al., 2021). Several studies suggested that XLGs regulate independent signaling pathways distinct from those that are regulated by the conventional G proteins. These may include nuclear signaling routes or cytoplasmic kinases distinct from GPA1-mediated cascades (Heo et al., 2012; Maruta et al., 2015; Hackenberg et al., 2016). Both GPA1 and XLGs have overlapping roles in regulating root development and immune signaling through partially distinct mechanisms (Pandey et al., 2008; Zhu et al., 2009; Maruta et al., 2015). The investigation into the interaction between the ubiquitin ligases PUB2/4 and XLGs indicated a potential shared functionality within specific pathways. Notably, the *xlg1, 2, 3* triple knockout mutants, the *pub4* mutant, and the *pub2/4* double mutant display observable deficiencies in cytokinin responses, stamen development, tapetum development, and male fertility (Wang et al., 2008). This indicates their involvement in cytokinin signaling and male fertility via ubiquitin-mediated regulation. Collectively, these findings highlight the integral role of XLGs in cytokinin-mediated development. XLGs, PUB2, and PUB4 are integral components of the intricate cytokinin signaling networks that govern numerous developmental and physiological processes (Wang et al., 2017).4 Roles of XLG protein in perception and response to environmental stresses.

In plants, receptors are essential for the perception of biotic and abiotic stress signals (**Table 1**); they further mediate cell-to-cell communication through efficient signaling (Jones and Dangl, 2006; Hématy et al., 2009).

**Table 1:** Involvement of heterotrimeric G proteins and subunits in plant abiotic stress response.

G protein	Function	Regulation of response	References
Drought Stress			Liang et al., 2017; Cantos et al., 2023; Takahashi et al., 2018; Sharma et al., 2023
GPA1	Positive regulator of drought response. Facilitates ABA-mediated stomatal closure	Regulates ion channels. Increases water use efficiency by reducing stomatal density	
AGB1	Negative regulator of drought response Impairs ABA signaling, downregulating ABA-responsive genes	Interacts with AtMPK6 to downregulate ABA-responsive genes	
AGG3	Uncertain involvement in drought stress response	Needs further exploration	
RGA1 (rice)	Negative regulator of ABA response and drought adaptation	Associated with ABA	
qPE9-1 (rice)	Negative regulator of ABA response and drought tolerance	Inhibits key transcription factors involved in ABA response	
RGB1 (rice)	Positive regulator of ABA-mediated drought stress response	Upregulates ABA biosynthesis	
PLD & PA	Dual role in responding to drought stress and stomatal movement Mediates ABA and ROS signaling	Activates PLD, inhibits RGS1 to promote GTPase activity of GPA1	
Ethylene-induced Closure	Mediates ethylene-induced stomatal closure Induces NADPH oxidase-dependent H2O2 in guard cells	Inactivates CTR1, leading to Ga-dependent H2O2 production	
Salinity Stress			Wang and Botella, 2022; Cui et al., 2020; Swain et al., 2019
Gα	Confers salinity tolerance when over-expressed. Regulates cell division and senescence under salt stress	Interacts with PLCd at C2 domain, activating signal transduction. Attenuates cell cycle regulation and cellular senescence
Gβ (AGB1)	Positively involved in the response to salt stress	Upregulates stress-related genes, involved in ion homeostasis
Gγ (RGG1, RGG2)	Participates in plant salt stress response	Upregulates antioxidant genes, enhancing antioxidant system
Other Abiotic Stresses			Suharsono et al., 2002; Sharma et al., 2023; Bai et al., 2018
RGA1	Mediates cold signal transduction	Interacts with COLD1 to activate Ca^2+^ channels and enhance GTPase activity
CsGG3.2	Regulates cold stress tolerance	Increases antioxidant enzyme activity (SOD, CAT, POD, GR) and reduces ROS accumulation
RGA1	Expression upregulated by heat stress	Mechanism in heat stress response remains unexplored
AGG3	Participates in regulation of oxidative stress and heavy metal stress tolerance	Enhances tolerance to cadmium stress in overexpression

### 4.1 Biotic stress responses

Plants perceive invading pathogens through the recognition of conserved molecular patterns and effector molecules using surface and intracellular receptors. The cell surface receptors, pathogen/microbe-associated molecular pattern (PAMP/MAMP) recognition receptors trigger pattern triggered immunity (PTI); and a class of nucleotide-binding leucine-rich repeat containing receptors (NLRs) activate effector triggered immunity (ETI) through recognition of pathogen effector proteins (Alberts et al., 2002; Hématy et al., 2009; Jones and Dangl, 2006).

Expression of PTI is mediated by cell surface–localized receptors including receptor-like kinases (RLKs) and receptor-like proteins (RLPs) phosphorylate receptor-like cytoplasmic kinases (RLCKs) such as BIK1, which in turn activate MAPK cascades and ROS production (Couto and Zipfel, 2016; Tang et al., 2017). PAMP/MAMP receptors, RLKs/RLPs, phosphorylate downstream RLCKs to mediated defense signaling following their activation (Torres et al., 2013). In *Arabidopsis*, approximately 610 RLKs are reported. Nearly 75% of these RLKs contain transmembrane and ectodomains (Shiu et al., 2004). The G protein is regulated by REGULATOR OF G-PROTEIN SIGNALING 1 (RGS1) for activation of downstream receptor kinases (Bender and Zipfel, 2018). XLG proteins also interact with RLKs. It is reported that an XLG protein directly binds to the receptor-like kinase FLS2 that recognizes the bacterial PAMP flagellin (flg22) (Liang et al., 2016).

*Arabidopsis* Gα subunit and G proteins are involved in mediating stomatal closure as a defense response (Lee et al., 2013). The interactions of G proteins with several other signaling proteins including mitogen-activated protein kinase (MAPKKKα), B-cell leukemia/lymphoma 2 protein (BcLCB2), *Capsicum annuum*mannose-binding lectins gene 1 (CaMBL1), *Nicotiana tabacum* leucine-rich protein 1 (NtLRP1), receptor-like cytoplasmic protein kinase (CaPIK1), ubiquitin ligase RING1 gene (CaRING1), heat shock protein 90 (HSP90), methionine sulfoxide reductase B2 gene (CaMsrB2), mitogen-activated protein kinase kinases 1 (MKK1) and vacuolar processing enzyme (VP) have been reported (Zhang et al., 2012).

XLG3 has been shown to enhance plant immunity against a diverse range of pathogens including bacterial pathogen *Pseudomonas syringae* pv. Tomato DC3000, fungal pathogen *Sclerotinia sclerotiorum*, and oomycetes pathogens *Phytophthora capsici*, *P. infestans*, and *P. Parasitica* (Li et al., 2022). XLG3 likely acts by modulating upstream signaling complexes or competing with GPA1 for Gβγ binding.XLG3 has been shown to play a crucial role in expressing immunity in several crops including rice (Biswal et al., 2022). XLG3 shares partial structural similarity with GPA1 enabling it to bind Gβγ dimers and modulate immune signaling. Therefore, XLG3 is possibly involved in signaling disease resistance response (Choudhury et al., 2013; Urano et al., 2016; Cantos et al., 2023).

G-proteins mediate changes in cytosolic Ca^2+^ levels, reactive oxygen species (ROS) production, and MAPK signaling (MAPK) signaling in response to biotic stress (Suharsono et al., 2002; Zhang et al., 2011). A recent study in *Nicotiana benthamiana* suggested that the PAMPs, flagellin (flg22) and chitin induce N-terminal phosphorylation of the NbXLG3 NLR protein (Li et al., 2022).These signals activate downstream defenses including stomatal closure, callose deposition, and hypersensitive response (HR) (Trusov et al., 2006).

The heterotrimeric G proteins are shown to contribute toward non-host resistance against fungal pathogens (Lee et al., 2013; Delgado-Cerezo et al., 2012; Zhang et al., 2025). Gα subunit is involved in the *NLR-*gene mediated ETI. The *gα* mutant showed reduced hypersensitivity, delayed defense gene expression, and suppressed H_2_O_2_ production reinforcing the Gα subunit’s role in disease resistance of rice against *Magnophorthe grisea* (Suharsono et al., 2002). The Gα subunit interacts with Rho GTPases linking defense signaling cascades via ROP/Rac-like GTPases in rice (Suharsono et al., 2002). The Gα and Gβ subunits directly or indirectly enhance jasmonate signaling pathway following infection of *Arabidopsis* with a necrotrophic pathogen (Trusov et al., 2006). The G protein subunits Gβ and Gγ but not Gα play an important role in plant defense against a variety of fungal pathogens including *Fusarium oxysporum*, *Alternaria brassicicola* and bacterial pathogen *Pseudomonas syringae* (Maruta et al., 2015). These findings emphasize the specialized roles of individual subunits in effector-triggered signaling. The Gβ, and Gγ dimer has been shown to directly interact with XLGs to mediate plant immunity especially in regulating ROS bursts and transcriptional responses. This partnership was demonstrated in *Arabidopsis* mutants lacking XLGs, Gα, Gβ, and Gγ. The mutants fail to mount effective defense responses, such as ROS bursts and gene activation (Maruta et al., 2015). Moreover, strong *xlg1xlg2xlg3* triple mutants were shown to be defective in PAMP-triggered MAPK activation and disease resistance revealing functional redundancy among all three XLGs in immunity (Wang et al., 2023).

In *Arabidopsis*, *XLG2* plays a key role in disease resistance (Zhu et al., 2009; Liang et al., 2016; Urano et al., 2016). The importance of XLG2 in enhancing resistance against the fungus *Magnaporthe oryzae* was revealed in the study of the *pen2–1 xlg2-1*double mutant which showed significantly higher penetration rates as compared to the *pen2–1* mutant. In contrast, the *pen2–1* and *xlg3–2* mutant plants exhibited a slight, non-significant increase in penetration rates as compared to the *pen2–1* mutant (Takahashi et al., 2018). XLG2, not XLG1 and XLG3, is involved in immunity of *Arabidopsis* against the bacterial pathogen *Pseudomonas syringae* (Zhu et al., 2009).

The *XLG2* and *XLG3* were induced in response to flg22 treatment and following infection with *Pseudomonas syringae* pv. Tomato (*Pst*) DC 3000 in the resistant accession (Liang et al., 2016). The *xlg2xlg3* double mutant displays compromised expression of *Pst* resistance and flg22-induced ROS suggesting additive effect of XLG3 with XLG2 (Maruta et al., 2015). XLG2 interacts with FLS2 and BIK1, regulates the accumulation of BIK1 for induction of immunity (Zhang et al., 2010; Liang et al., 2016).

Wang et al. (2023) demonstrated that XLG1, in conjunction with XLG2 and XLG3, is essential for full activation of MAPKs in response to PAMPs. The triple *xlg1xlg2xlg3* mutant showed severely compromised MAPK activation (MKK4/MKK5 → MPK3/MPK6) and pathogen resistance. These XLGs directly interact with MAPKKK3/5 and are crucial for PAMP-triggered immunity.

### 4.2 Abiotic stress responses

Plant growth and development are impeded by abiotic stresses such as temperature, drought and salinity (Zhu, 2016; **Table 1**).

In rice, four putative XLGs (PXLGs), namely PXLG1, PXLG2, PXLG3, and PXLG4, were investigated for their roles in regulating yield components and stress tolerance through the study of CRISPR/Cas9-induced *xlg* mutants. It was observed that PXLG3 and PXLG4 have strong involvement in salt and drought stress signaling (Cui et al., 2020; Zhang et al., 2015). RGB1, the rice Gβ subunit, contributes to plant architecture by inducing semi-dwarf phenotypes and enhances salt tolerance by modulating stress-responsive signaling pathways (Li et al., 2016). RGB1, along with qPE9-1, has been identified as a regulator of abscisic acid and a contributor to drought response (Zhang et al., 2015). Swain et al. (2019) demonstrated that the over expression of *RGB1* and *RGG1* genes imparts multiple stress tolerance in rice by inducing the expression of stress-responsive genes. In tomato, overexpression of *LeGPA1* showed elevated oxidase and catalase activity, enhancing tolerance to cold (Guo et al., 2020). It suggests that LeGPA1 positively regulates ROS-scavenging mechanisms under low-temperature stress conditions.

Together, these findings indicate that heterotrimeric G-protein components, including XLGs, Gα, Gβ, and Gγ subunits, act as central hubs that integrate hormonal and environmental signals to confer adaptive responses under abiotic stress conditions. Their roles in regulating gene expression, ROS detoxification, and hormone signaling position them as promising targets for developing stress-resilient crops.

### 4.3 Crosstalk of XLG proteins with hormonal and secondary signaling pathways

XLGs integrate not only in the canonical G-protein complex but also interact with multiple hormonal and secondary signaling pathways that orchestrate plant development and stress responses. The loss-of-function *Arabidopsis xlg2* and *xlg3* mutants exhibit reduced stomatal closure and diminished ABA sensitivity under drought, pointing to their involvement in ABA‐mediated stress signaling (Urano et al., 2016). Additionally, jasmonic acid (JA) responsive gene, such as *PDF1.2* gene expression is attenuated in G-protein mutants, indicating that XLG–Gβγ may regulate JA‐mediated defense gene activation pathways (Maruta et al., 2021). Heterotrimeric G-proteins integrate multiple upstream signals from receptor-like kinases, thereby coordinating plant immune responses (Liu et al., 2013).

New evidence from rice transcriptomic analyses reveals synergistic upregulation of JA biosynthesis genes, e.g. *OsAOS2* and *OsOPR1*, following combined ABA and pest stress, suggesting strong ABA/JA crosstalk in defense regulation (Li et al., 2022). In *Arabidopsis*, ABA receptors, e.g. PYR/PYL family, interact with JAZ/JAZ–MYC2 modules, modulating JA signaling outputs during stress responses. Abscisic acid receptors are involved in jasmonate signaling (Sharma et al., 2023).

Beyond hormonal crosstalk, XLGs also appear to modulate secondary messengers such as Ca²^+^ and reactive oxygen species (ROS). Upon PAMP treatment with either flg22 or chitin, XLG3 undergoes N-terminal phosphorylation in *Nicotiana benthamiana*, coinciding with robust ROS burst and calcium fluxes (Li et al., 2022). Moreover, XLGs form complexes with MAPKKKs/MAPKs and activate upstream of MKK4/5 and MPK3/6 to amplify defense signaling (Biswal et al., 2022; Maruta et al., 2021).

## 5 Potential applications of XLG proteins in crop stress engineering

Given their dual roles in plant development and stress adaptation, XLG proteins emerge as promising targets for bioengineering enhance crop resilience. In rice, CRISPR/Cas9-mediated editing of *PXLG1–4* genes altered plant architecture and improved salinity tolerance, establishing their contributions to yield and stress responses (Cui et al., 2020). Moreover, modulation of the rice Gβ subunit RGB1, which interacts functionally with XLGs, influences both salt stress tolerance and plant architecture via ABA signaling pathways (Li et al., 2016). Over expression of RGB1 and RGG1 enhances drought, salinity, and oxidative stress tolerance, demonstrating their positive regulatory roles on mechanisms that provide tolerance against these abiotic stresses (Swain et al., 2019).

In tomato, overexpression of *LeGPA1* enhances cold stress tolerance by boosting antioxidant enzyme activity and stress-responsive gene expression suggesting that targeting XLG signaling components could similarly improve abiotic stress tolerance (Guo et al., 2020). Combining marker-assisted introgression of favorable XLG alleles from wild germplasm or through allele swapping by genome editing could accelerate crop breeding for developing climate-resilient cultivars (Fan et al., 2024; Washington, 2024).

## 6 Conclusion

Emerging studies revealed that *XLGs* are not only involved in regular plant development but also associated with PAMP-triggered immunity (Lou et al., 2020; Biswal et al., 2022). *XLG3* supplements the *XLG2* gene function and enhances the disease resistance response (Heo et al., 2012; Liang et al., 2017). In recent years, investigations of the XLGs and G proteins primarily in *Arabidopsis* and rice revealed the regulatory roles of XLGs in providing plants with both biotic and abiotic stress tolerance (Takahashi et al., 2018; Yang et al., 2021; Cantos et al., 2023). However, the underlying mechanisms of XLG-mediated signaling pathways in plants still need to be explored. The underlying mechanisms for the upstream activation of G protein signaling in crops that lack RGS proteins need to be investigated. It indicates the possible presence of unknown regulators with GAP activity (Urano et al., 2016). The mechanisms of signal perception by XLGs also need to be explored, particularly the functions of PLSs with GEF activity and their potential roles in the function of GPCR-like receptors (Zhang et al., 2025; Taddese et al., 2014). The downstream signaling pathways of plant G proteins also need to be studied (Urano et al., 2013; Maruta et al., 2015). Future studies focusing on XLGs and their interactors will provide the mechanistic role of the XLG-mediated regulation of biological processes related to plant development and adaptation of plants to environmental stresses.
